# The epigenetic modifier JMJD6 is amplified in mammary tumors and cooperates with c-Myc to enhance cellular transformation, tumor progression, and metastasis

**DOI:** 10.1186/s13148-016-0205-6

**Published:** 2016-04-14

**Authors:** Olga Aprelikova, Kenny Chen, Lara H. El Touny, Constance Brignatz-Guittard, Justin Han, Tinghu Qiu, Howard H. Yang, Maxwell P. Lee, Min Zhu, Jeffrey E. Green

**Affiliations:** Laboratory of Cancer Biology and Genetics, National Cancer Institute, National Institutes of Health, Building 37, Room 4054, 37 Convent Dr., Bethesda, MD 20892 USA

**Keywords:** Mammary cancer, Myc, JMJD6, Copy number variants, Epigenetics, Tumor progression

## Abstract

**Background:**

Oncogene overexpression in primary cells often triggers the induction of a cellular safeguard response promoting senescence or apoptosis. Secondary cooperating genetic events are generally required for oncogene-induced tumorigenesis to overcome these biologic obstacles. We employed comparative genomic hybridization for eight genetically engineered mouse models of mammary cancer to identify loci that might harbor genes that enhance oncogene-induced tumorigenesis.

**Results:**

Unlike many other mammary tumor models, the MMTV-Myc tumors displayed few copy number variants except for amplification of distal mouse chromosome 11 in 80 % of the tumors (syntenic to human 17q23-qter often amplified in human breast cancer). Analyses of candidate genes located in this region identified JMJD6 as an epigenetic regulatory gene that cooperates with Myc to enhance tumorigenesis. It suppresses Myc-induced apoptosis under varying stress conditions through inhibition of p19ARF messenger RNA (mRNA) and protein, leading to reduced levels of p53. JMJD6 binds to the p19ARF promoter and exerts its inhibitory function through demethylation of H4R3me2a. JMJD6 overexpression in MMTV-Myc cell lines increases tumor burden, induces EMT, and greatly enhances tumor metastasis. Importantly, we demonstrate that co-expression of high levels of JMJD6 and Myc is associated with poor prognosis for human ER+ breast cancer patients.

**Conclusions:**

A novel epigenetic mechanism has been identified for how JMJD6 cooperates with Myc during oncogenic transformation. Combined high expression of Myc and JMJD6 confers a more aggressive phenotype in mouse and human tumors. Given the pleiotropic pro-tumorigenic activities of JMJD6, it may be useful as a prognostic factor and a therapeutic target for Myc-driven mammary tumorigenesis.

**Electronic supplementary material:**

The online version of this article (doi:10.1186/s13148-016-0205-6) contains supplementary material, which is available to authorized users.

## Background

Tumorigenesis is a multistep process involving the accumulation of genetic aberrations. Initiating oncogenic alterations promote the selection of additional genetic and epigenetic changes that favor transformation, tumor growth, and metastases. Initiating oncogenic alterations in untransformed cells in most, if not all cases, trigger cellular safeguard mechanisms that induce cellular senescence (e.g., Ras) or apoptosis (e.g., Myc, E2F1, or E1A). Therefore, oncogene-induced tumorigenesis generally requires cooperating genetic events to overcome these safeguard mechanisms.

It has been firmly established that many genetically engineered mouse models (GEMs) of mammary cancer are valuable systems to dissect tumorigenic pathways that may involve multiple genetic aberrations. Many models have been designed to mimic human breast cancer by either overexpression of known oncogenes (Myc, Ras, Wnt, PyMT, Erbb2) or deletion of tumor suppressor genes (BRCA1/2, p53, and Rb) [1, 2]. For example, several mouse models of breast cancer, including MMTV-PyMT or WAP-Myc, express markers associated with human luminal-type breast cancer [3]. Murine models (C3(1)-Tag or BRCA1 deficiency together with p53 mutation) allow tumor development with a characteristic basal-like phenotype [1, 3–5]. On the other hand, some other models show mixed features and high levels of heterogeneity [3]. Unlike human breast cancers, many mouse models of mammary cancer are based upon the induction of a specific oncogenic event through the overexpression of relevant oncogenes or inactivation of tumor suppressor genes, leading to the evolution of oncogene-specific secondary pathways.

A predominant mechanism leading to additional genetic alterations required for progression of tumorigenesis appears to be related to changes in genome copy number variants (CNV). A recent study demonstrated that 22 % of the haploid genome in breast cancer is affected by chromosome rearrangements [6], thus indicating that CNVs are the major contributor to the accumulation of additional genetic changes during tumor progression.

Array comparative genomic hybridization (CGH) has been a powerful tool to identify chromosomal regions that may harbor amplified oncogenes or deleted tumor suppressor genes. These techniques combined with gene expression analysis, revealed a significant correlation between human and multiple GEM models of mammary gland tumors [3, 7, 8]. Recently 662 regions of chromosomal aberrations conserved between human and mouse breast cancers were identified [8]. These studies allow not only the identification of novel drivers of tumorigenesis but also find supporting genetic alterations necessary for promoting cellular transformation and tumor progression.

We used array CGH to identify CNVs in eight genetically engineered mouse models of mammary cancer to find recurrent CNVs that potentially could harbor critical genes enhancing oncogene-induced transformation. We determined that the distal region of chromosome 11 was amplified in 80 % of MMTV-Myc-driven mouse mammary gland tumors. This locus is syntenic to human chromosome 17q23-qter, a region that is often amplified in human breast cancers. We hypothesized that gene(s) located in this locus are critical for Myc-induced tumor development and progression. Myc is commonly amplified in many cancers of different origins. Importantly, Myc is overexpressed in 25–30 % of all breast cancer cases [9–12].

Further functional analyses of several candidate genes overexpressed in the mouse 11q locus revealed that the epigenetic modifier JMJD6 is able to inhibit Myc-induced apoptosis, which is critical for tumor progression. While Myc-triggered cell death may involve several pathways, the predominant responder to aberrant induction of Myc in primary cells is p19ARF and upregulation of p53. We found that JMJD6 represses p19ARF, at least in part, by demethylation of Arg3 of histone H4 associated within the p19ARF promoter. Therefore, JMJD6 amplification may cooperate with Myc to enhance neoplastic transformation of primary epithelial cells. Additionally, when overexpressed in MMTV-Myc-driven tumor cells that lack the 11q amplification, JMJD6 induces EMT, increases cell migration and invasion in vitro, and stimulates tumor growth in vivo. Most importantly, JMJD6 dramatically increased lung metastatic colonization of these otherwise non-metastatic cells. Bioinformatics analyses of human breast cancer tumors revealed a significant decrease in survival of patients with ER+ tumors when Myc and JMJD6 were highly expressed together as compared to high Myc expression alone.

Identification of JMJD6 as a gene that cooperates with Myc to enhance tumorigenesis could provide a novel therapeutic target for breast and other cancers where Myc is an essential driver of tumorigenesis, since to date no successful therapies directly targeting Myc have been developed.

## Results

### Identification of a chromosome 11 amplicon as the major genomic alteration in MMTV-Myc mammary tumors

**Fig. 1:**
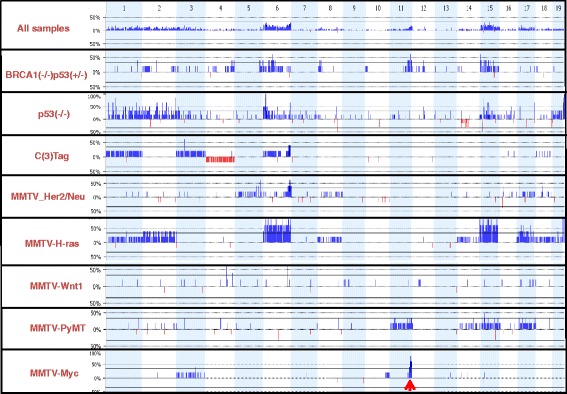
DNA copy number analysis. Array CGH analysis of mouse mammary gland tumors from eight genetically engineered models of breast cancer. 5–6 tumors were used for each model. The threshold line is drawn at 35 % of samples. Genomic regions of significant gains are shown in *blue* and significant losses are shown in *red. Arrow* indicates the amplification of distal mouse chromosome 11

We performed comparative genomic hybridization (CGH) of mammary gland tumors from eight genetically engineered mouse models to identify genomic loci containing genes with altered expression that potentially cooperate with oncogenes or suppressor genes in promoting tumorigenesis. On average, DNAs from 5–6 non-necrotic tumor samples from each model were analyzed by array CGH using the Agilent 44K array platform. Spleen DNA from the background strain served as the corresponding control. Our CGH results identified previously reported copy number variants (CNVs) and chromosomal aberrations in these models [8, 13–16], validating the results. In addition to the amplification of distal chromosome 6 in C3(1)-Tag model that we had previously reported [17], we identified amplification of chromosome 6 in four additional tumor models, gain of partial or whole chromosome 15 in four models, and loss of chromosome 4 in some C3(1)-Tag-driven tumors (Fig. 1), consistent with prior reports [8, 13, 15, 16, 18]. Interestingly, 80 % of MMTV-Myc-driven tumors exhibited minimal genomic changes except for amplification of the distal part of chromosome 11. We also observed this amplification in some MMTV-PyMT and BRCA−/−; p53+/− tumors as have been previously reported [8, 13, 15, 16]. However, these two models also exhibited other large regions of chromosomal amplifications. Therefore, we chose to focus on the MMTV-Myc model since the chromosome 11 amplification region likely contains genes required for Myc tumorigenesis. Importantly, this region is syntenic to human chromosome 17q23-qter, which is often amplified in human breast cancer patients [19–22].

### Identification of genes overexpressed in the chromosome 11 amplicon in MMTV-Myc mammary tumors

**Fig. 2:**
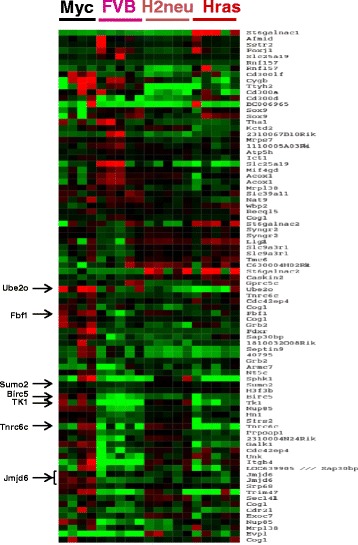
Gene expression microarray analysis for chromosome 11 amplified region. The heatmap shows the differential gene expression in mammary gland tumors from MMTV-Myc transgenic mice with chromosome 11 amplification versus MMTV-Her2, or MMTV-HRas tumors lacking the chromosome 11 amplification, or normal lactating mammary glands from FVB/N mice. Genes labeled in *red* are expressed at higher than median levels and genes labeled in *green* are expressed at lower than median levels. Genes selected for further validation are indicated on the *left side*

To gain more insight into the function of the genes located in this region, we determined the minimal region that was amplified in chromosome 11 across the MMTV-Myc tumors and found that it contained 246 genes and one microRNA. The exact chromosomal coordinates for those genes are presented in Additional file 1: Figure S1. To identify the genes that had higher expression in MMTV-Myc tumors compared to normal mammary gland tissue or tumors from other models that did not contain the chromosome 11 amplification, we performed microarray analysis using RNA from normal glands as well as several mammary gland tumors derived from MMTV-HRas and MMTV-Her2/Neu mice (Fig. 2). Using these data together with a literature-based screen of known gene functions, we selected seven genes from the chromosome 11 candidate interval for further validation (FBF1, Ube2o, TK1, Birc5, Sumo2, Tnrc6c, and JMJD6).

To identify potential candidate cell lines derived from MMTV-Myc tumors for further in vitro studies, we performed CGH analysis of several cell lines and found that the Myc83 cell line harbors the chromosome 11 amplicon, as previously observed [23], while the 88CT1 cell line has relatively few CNVs without amplification of the chromosome 11 locus (Additional file 1: Figure S2A). We also found increased expression levels of selected genes (JMJD6, Tnrc6c, and Ube2o) by RT-qPCR in the Myc83 cells compared to 88CT1 cells, consistent with the status of the chromosome 11 amplification (Additional file 1: Figure S2B).

### Identification of JMJD6 as a gene that suppresses Myc-induced apoptosis

Since Myc expression increases apoptosis in primary cells, which is a major response preventing full transformation of cells by Myc alone, we tested the hypothesis that the increased expression of candidate genes would suppress Myc-induced cell death, whereas depletion of any of the seven selected genes would increase Myc-induced cell death in vitro. Myc-induced apoptosis in many cases requires an intact p53 pathway. Sequence analysis confirmed the wild-type status of p53 in both Myc83 and 88CT1 cells. However, etoposide treatment of these cell lines revealed that Myc83 responded to etoposide with a robust increase in p53 and p21 protein levels, while 88CT1 had a much lower expression of p53 (possibly because of the amplification of MDM2 in these cells, as determined by CGH analysis). Therefore, for the primary apoptosis screen, we chose Myc83 cells that express high constitutive levels of c-Myc and contain the chromosome 11 amplification. The limitation of this model is that one cannot conclude that cell death is Myc-dependent or whether the tested genes have a more general effect on cell viability.

**Fig. 3:**
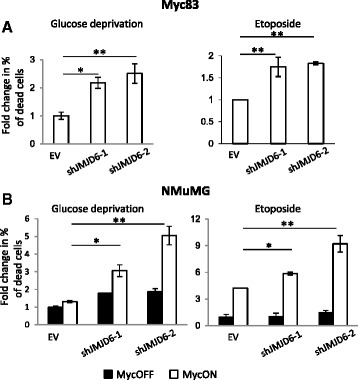
Analysis of cell death induced by glucose deprivation or etoposide treatment in cells with JMJD6 knock-down. **a** Myc83-derived cell lines (parental line from a MMTV-Myc tumor) with stable expression of two independent shRNAs targeting JMJD6 or with empty vector (EV) control were treated with 100 μM etoposide or grown in glucose-free media for 20 h. Cell death was measured using CytoTox-Glo reagent. The experiments were repeated 3 times and the percentage of dead cells in each experiment was expressed relative to control (EV) cells. **b** NMuMG cells with MycER™ and shJMJD6 expression were first treated with 150 nM 4-OHT (MycON) or ethanol (MycOFF) for 24 h to activate Myc and then treated as in **a** *Black bars*—MycOFF (ethanol-treated cells), *open bars*—MycON (4-OHT-treated cells). Results were normalized to EV control with MycOFF. *p < 0.05, **p < 0.01

To clarify this question, we established another model using normal murine mammary gland (NMuMG) epithelial cells with inducible expression of MycER™ where c-Myc is fused in frame with the mutated estrogen receptor-binding domain which makes it refractory to beta-estradiol but that can be activated by the addition of 4-hydroxytamoxifen [24]. First, we tested five different small hairpin RNA (shRNA) constructs for each gene and selected those that provided at least a 50 % reduction in gene expression. These shRNAs were then stably expressed in Myc83 cells and NMuMG-MycER^TM^ cells and the resultant cells were treated with etoposide or glucose deprivation. As shown in Fig. 3 and Additional file 1: Figure S3, the most consistent increase in Myc-dependent cell death in both cell types was obtained after depletion of JMJD6. The efficiency of JMJD6 knock-down by two shRNAs is shown in Additional file 1: Figure S4. Several other genes showed promising results in cooperation with Myc in MNuMG cells (FBF1) and others in Myc83 cells (Sumo2), which may reflect limited cell type-specific functions of these genes. We, therefore, focused on exploring the cooperation of JMJD6 with c-Myc in cellular transformation, tumor progression, and metastases.

### The anti-apoptosis effect of JMJD6 is dependent upon JMJD6 enzymatic activity

**Fig. 4:**
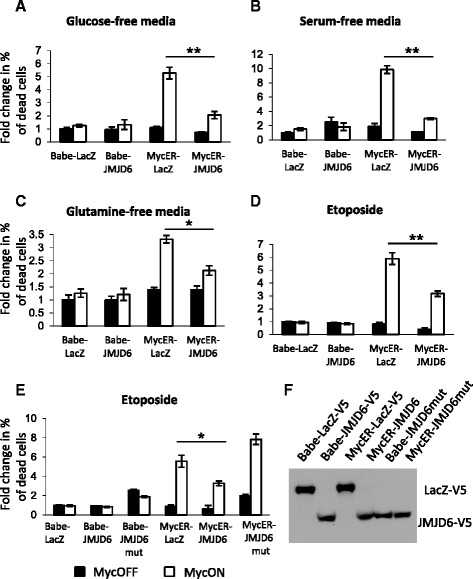
Overexpression of wild-type, but not mutated, JMJD6 suppresses Myc-induced cell death in response to different stress conditions. NMuMG cells stably expressing MycER^TM^ or empty pBabe vector were transduced with JMJD6-V5 or LacZ-V5 control. Cells were treated with 4-OHT or ethanol as in Fig. 3 and placed in glucose-free (**a**), serum-free (**b**), or glutamine-free (**c**) media or treated with etoposide (**d**) for another 20 h. Cell death was measured as in Fig. 3. *Black bars*—MycOFF, *open bars*—MycON. **e** Catalytically inactive JMJD6H187A (JMJD6mut) is not able to suppress Myc-induced cell death. *p < 0.05, **p < 0.01. **f** Western blot analysis shows equal ectopic expression of JMJD6 or mutated JMJD6 in NMuMG cells with our without Myc expression

JMJD6 is an enzyme with pleiotropic functions that has been recently implicated in the breast, and some other cancers where high expression of JMJD6 was an indicator of poor prognosis [25–28]. We further validated our model system using NMuMG cells with constitutive overexpression of JMJD6 and an inducible c-Myc. As expected, ectopically expressed JMJD6 was localized to cell nuclei whereas control cells exhibited ectopic cytoplasmic expression of LacZ (Additional file 1: Figure S5A). The overall levels of JMJD6 in transfected cells were close to physiological levels observed in cells with amplified chromosome 11 (about 3-fold over control cells, Additional file 1: Figure S5B). Cells expressing high levels of c-Myc proved to be sensitive to multiple stress conditions, including depletion of nutrients or growth factors, or treatment with DNA-damaging agents, leading to cell death. C-Myc induction in NMuMG cells followed by exposure to four different stress conditions resulted in a significant increase in cell death, which was reduced by co-expression with JMJD6 (Fig. 4a–d).

JMJD6 is an iron- and 2-oxoglutarate-dependent dioxygenase with the capability to hydroxylate lysine residues in histone and non-histone proteins [26, 29, 30]. Lysine hydroxylation of RNA splicing factors results in production of differentially spliced pre-messenger RNA (mRNA) molecules [31], while hydroxylation of histone proteins may result in changes in transcriptional regulation of targeted gene expression [32]. In order to determine whether the enzymatic activity of JMJD6 is necessary for the inhibition of Myc-induced cell death, we mutated His187 to Ala in the iron-coordinating center of the enzyme that has previously been showed to inactivate JMJD6 and demonstrated that mutant JMJD6 does not suppress cell death (Fig. 4e). Western blotting confirmed that the levels of mutated protein expression were similar to the expression of the wild-type JMJD6 (Fig. 4f).

**Fig. 5:**
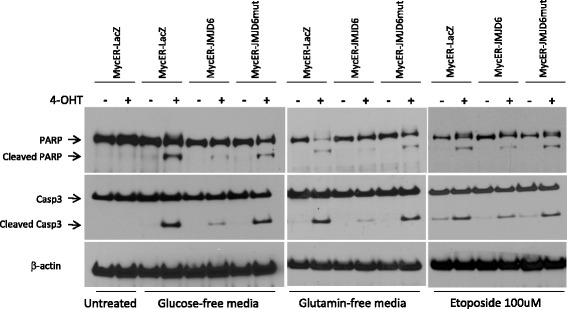
Western blot analysis of apoptotic markers in Myc-induced cells in the presence of wild-type or mutated JMJD6. NMuMG cells were treated with 150 nM 4-OHT to induce Myc or ethanol and placed in glucose- or glutamine-free media or treated with 100 mM of etoposide. Expression of cleaved PARP and cleaved caspase 3 was determined by Western blot demonstrating reduced levels in the presence of wild-type, but not mutant, JMJD6

To further prove that high levels of wild-type JMJD6, but not its mutated form, inhibit cell death, we examined protein levels of cleaved caspase 3 and PARP (markers of apoptosis) following Myc induction and exposure to different stress conditions. While control cells showed robust cleavage of both enzymes upon c-Myc induction, cleavage was clearly diminished when cells co-expressed wild-type JMJD6 (Fig. 5). However, the expression of mutant JMJD6 had a minimal effect on suppressing caspase 3 and PARP cleavage, which remained similar to levels observed in control cells (Fig. 5).

In order to understand mechanisms by which JMJD6 inhibits Myc-induced cell death, we first analyzed its effect on Myc protein levels and cellular localization. Additional file 1: Figure S5A shows that MycER™ or endogenous levels of c-Myc were not compromised by overexpression of JMJD6. Also, in the presence of 4-hydroxytamoxifen, ectopically expressed MycER™ re-localizes to the cell nucleus similar to control cells without JMJD6 overexpression (Additional file 1: Figure S6B).

### Identification of mechanisms contributing to JMJD6 inhibition of Myc-induced apoptosis

**Fig. 6:**
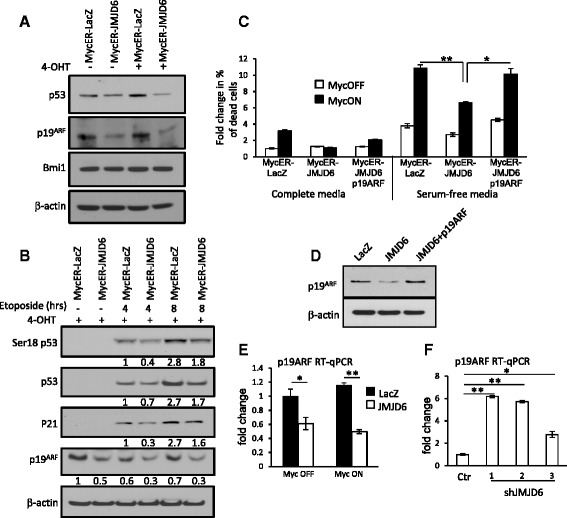
JMJD6 overexpression reduces p53 and p19ARF levels. **a** Immunoblotting of NMuMG cells with ectopic expression of JMJD6 or LacZ control shows decreased protein levels of p53 and p19ARF in cells with or without Myc induction. **b** Cells were stimulated with 4-OHT to induce Myc and then treated with 100 mM etoposide for 4 and 8 h to analyze p53 induction. Immunoblots show lower levels of p53 or Ser18-phosphorylated p53 as well as p19ARF and p21 in cells expressing JMJD6. Intensity of each band normalized to β-actin was obtained using ImageJ software. **c** Overexpression of p19ARF in the presence of JMJD6 restores Myc-induced cell death. NMuMG cells expressing MycER™ together with JMJD6, LacZ, or JMJD6 plus p19ARF were treated with 4-OHT (MycON) or ethanol (MycOFF) for 24 h and placed in serum-free media for another 24 h. Percentage of dead cells was measured with CytoTox-Glo reagent. *Black bars*—MycON, *open bars*—MycOFF. **d** Western blot analysis of NMuMG cells with ectopic overexpression of p19ARF. **e** RT-qPCR analysis shows that overexpression of JMJD6 in NMuMG cells inhibits p19ARF transcription. *Black bars*—NMuMG cells expressing LacZ control, *open bars*—cells expressing wild-type JMJD6. **f** Knock-down of endogenous JMJD6 with three different shRNAs increases mRNA levels of p19ARF

We then sought to establish which pathways involved in Myc-induced cell death are inhibited by elevated levels of JMJD6 expression. In many cellular models, Myc-induced apoptosis stimulates induction of p19ARF that sequesters MDM2 ubiquitin ligase, allowing for the upregulation of p53 that activates apoptotic pathways [33–35]. We first found that in NMuMG cells, JMJD6 downregulates the endogenous levels of p19ARF and p53, with or without c-Myc induction (Fig. 6a). It has been known that p19ARF may be suppressed by Bmi1 protein; however, we found no difference in Bmi1 protein levels in cells with high expression of JMJD6 (Fig. 6a). We also analyzed p19ARF and p53 induction after treatment with the DNA-damaging agent etoposide in the presence of JMJD6. JMJD6 expression resulted in lower levels of p19ARF, p53 total protein, and its Ser18 phosphorylated form as well as p21 proteins at all time points after etoposide treatment, compared to LacZ control (Fig. 6b).

Our finding that JMJD6 may reduce p19ARF stimulation by Myc led us to determine whether the blunting effect of JMJD6 on Myc-induced cell death is p19ARF dependent. We overexpressed p19ARF protein in cells with JMJD6 and the inducible MycER^TM^ and determined levels of cell death after starving cells in serum-free conditions. Indeed, increased levels of p19ARF protein rescued Myc-induced apoptosis in cells with high JMJD6 expression, demonstrating that p19ARF suppression by JMJD6 contributes to the alleviation of cell death (Fig. 6c, d).

To further confirm that high expression of JMJD6 would correlate with decreased expression of p19ARF in vivo, we analyzed expression of these two genes in mouse MMTV-Myc mammary gland tumors. This experiment also showed robust negative correlation between JMJD6 and p19ARF (Additional file 1: Figure S7).

### JMJD6 reduces p19ARF expression through histone H4 modifications on the p19ARF promoter region

JMJD6 is involved in post-translational modifications of non-histone and histone proteins [29, 30, 32, 36]. However, when JMJD6 regulates histone modifications, it modifies mRNA levels of the target gene. To discriminate which of the pathways is involved in p19ARF silencing by JMJD6, we studied p19ARF transcript levels in the presence of high JMJD6 expression. We clearly observed that JMJD6 overexpression significantly reduces mRNA levels of p19ARF (Fig. 6e), while three different JMJD6 shRNA constructs significantly increased p19ARF mRNA when expressed in NMuMG cells (Fig. 6f).

**Fig. 7:**
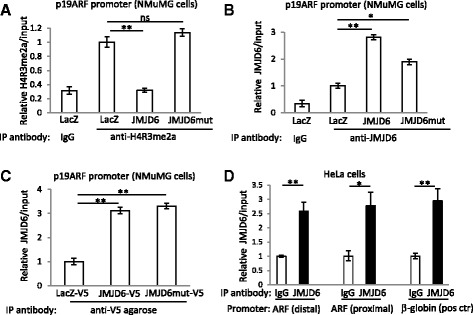
JMJD6 binds to the p19ARF promoter and decreases histone H4R3 asymmetric dimethylation of the p19ARF promoter. ChIP analysis of the p19ARF promoter in NMuMG cells expressing LacZ, JMJD6, or JMJD6 mutant using immunoprecipitation with **a** anti-H4R4me2a, **b** anti-JMJD6 antibody, **c** V5-conjugated agarose. **d** ChIP analysis of HeLa cells shows binding of endogenous JMJD6 to the human p14ARF promoter. Two different primer sets (distal and proximal) were used for qPCR after immunoprecipitation. Primers for human β-globin gene promoter were used as a positive control

Previously published data suggest that JMJD6 is able to remove methyl groups from symmetrically (H4R3me2s) or asymmetrically (H4R3me2a) methylated arginines [32, 36]. This allows JMJD6 to act as a transcriptional activator or repressor, as H4R3me2s modification is associated with repressed chromatin [37, 38] and H4R3me2a is a mark for transcriptionally active chromatin [37, 39, 40]. To this end, we performed chromatin immunoprecipitation experiments to compare the amounts of activating H4R3me2a associated with the p19ARF promoter in cells expressing high levels of JMJD6 versus cells with low expression of JMJD6. First, we found that control cells expressing LacZ or mutated JMJD6 have a significantly higher presence of H4R3me2a within the p19ARF promoter than cells with overexpression of JMJD6 (*p* = 0.01) (Fig. 7a). Furthermore, chromatin immunoprecipitation with antibodies specific for JMJD6 showed that JMJD6 as well as mutated JMJD6 protein is bound to the p19ARF promoter (Fig. 7b). We also analyzed JMJD6 effect on p16 and found no expression of p16 in control or JMJD6-overexpressing NMuMG cells by RT-qPCR or Western blotting. Therefore, to prove specificity of anti-JMJD6 antibody in ChIP assay, we used p16 promoter primers and found no binding of JMJD6 to p16 promoter (Additional file 1: Figure S8). In addition, since control LacZ, JMJD6, and JMJD6 mutants were fused with C-terminal V5 tag, we performed ChIP analysis using V5-conjugated agarose and confirmed higher abundance of JMJD6 and JMJD6 mutant binding to the p19ARF promoter compared to LacZ control (Fig. 7c).

Since endogenous mouse JMJD6 is poorly recognized by the antibody available for ChIP studies, we decided to test human HeLa cells to analyze whether endogenous JMJD6 in these cells is found on the ARF promoter. We tested the human p14ARF promoter with two primer sets using a human JMJD6 antibody for ChIP and found higher levels of JMJD6 associated with the ARF promoter compared to immunoprecipitation with a non-specific IgG (Fig. 7d). In summary, we found that in non-tumorigenic mouse epithelial cells, JMJD6 may collaborate with c-Myc to initiate tumor formation by suppressing Myc-induced cell death by inhibiting transcriptional expression of the p19ARF tumor suppressor protein.

### JMJD6 promotes Myc-induced tumor formation

**Fig. 8:**
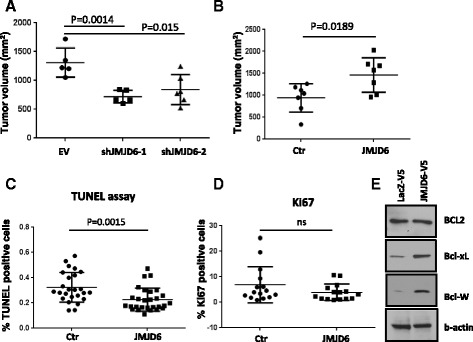
JMJD6 promotes tumor growth of cells derived from MMTV-Myc mammary gland tumors. **a** 10^6^ Myc83 cells (with amplification of the chromosome 11 locus containing JMJD6) stably expressing empty vector (EV) or two independent shRNAs targeting JMJD6 were injected into mammary fat pads of FVB/N mice and tumors were measured over the next month. **b** 5 × 10^5^ 88CT1 cells (no chromosome 11 amplification) with stable expression of LacZ control or wild-type JMJD6 were surgically implanted into mammary fat pads and tumor growth was measured over the next 20 days. **c** Tumors from **b** were formalin fixed and sectioned and the percentage of dead cells was analyzed by TUNEL assay. **d** Tumors from **b** were immunostained with anti-Ki67 antibodies and signal intensity was quantitated by ImageJ software. **e** Western blot analysis of pro-survival genes in 88CT1 cells expressing JMJD6

We sought to determine to what extent established mammary tumors remain dependent on JMJD6 expression for tumor maintenance and an aggressive phenotype. We first knocked down JMJD6 in Myc83 cells with elevated JMJD6 expression using two different shRNAs and found a significant delay in tumor formation compared to control cells when injected into mouse mammary fat pad (Fig. 8a). To show the reciprocal effect of increased JMJD6 expression in low JMJD6-expressing cells, overexpression of JMJD6 in 88CT1 cells resulted in significantly larger tumors compared to LacZ control-expressing cells (Fig. 8b). The tumors derived from 88CT1 cells ectopically expressing JMJD6 exhibited significantly less apoptosis by TUNEL assay (Fig. 8c) than control cells but no difference in expression of the cell proliferation marker Ki67 (Fig. 8d).

Interestingly, we demonstrated that 88CT1 cells established from MMTV mice have a compromised p53 pathway, possibly resulting from amplification of MDM2 (gain of 8 copies) as determined by array CGH. We found that the reduction in the percentage of apoptotic cells in 88CT1 cells overexpressing JMJD6 was associated with elevated levels of the Bcl2 anti-apoptotic family members Bcl-xl and Bcl-w (Fig. 8e) that were previously shown to inhibit Myc-induced cell death [41–43].

### JMJD6 enhances cell migration, invasion, and metastases

MMTV-Myc mammary gland tumors usually produce well-differentiated tumors with a relatively long latency and relatively few metastases [12, 44, 45], implying that additional genetic alterations are required for a more aggressive phenotype. However, several observations raise the possibility that elevated expression of Myc protein may inhibit cellular migration, invasion, and metastasis formation in mouse xenograft models of breast cancer [46]. We therefore investigated whether JMJD6 might enhance migration, invasion, and metastases of MMTV-Myc mammary tumor cells.

**Fig. 9:**
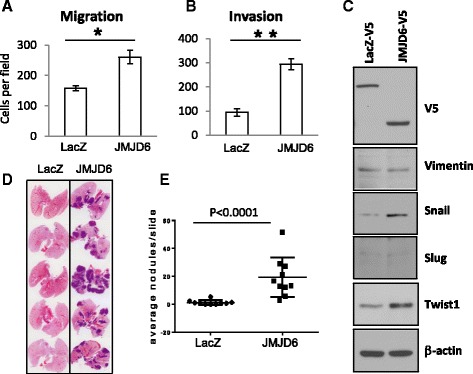
JMJD6 increases the metastatic propensity of c-Myc-expressing cells. Transwell migration (**a**) and invasion (**b**) assays of 88CT1 cells overexpressing JMJD6 compared to LacZ control (*p < 0.05, **p < 0.01, respectively). **c** Western blot analysis of EMT markers in the cells used in **a** and **b** shows increased expression of Snail and Twist1 in cells with high expression of JMJD6. **d** Lung colonization in vivo. 5 × 10^5^ cells used in **a** and **b** were injected by tail vein into FVB/N mice. After 20 days, the lungs were formalin fixed and stained with H&E. **e** Quantitation of metastatic nodules per lung of mice shown in **d**

Ectopic expression of JMJD6 in 88CT1 cells resulted in a 2–3-fold increase in motility and invasion compared to non-JMJD6-expressing cells using the Boyden chamber assays (Fig. 9a, b) and was associated with increased expression of the EMT markers Snail and Twist1, while others, vimentin and Slug, remained unchanged (Fig. 9c). To test the metastatic propensity of 88CT1 cells overexpressing JMJD6, we injected those cells or LacZ control cells via mouse tail vein and analyzed lung sections 21 days later. Control cells produced very few and in many cases no metastatic nodules while cells with elevated expression of JMJD6 showed a dramatic 20-fold increase in the number of lung colonies (*p* < 0.0001, Fig. 9d, e). These results demonstrate that JMJD6 contributes to Myc-induced mammary gland tumor maintenance and confers a highly metastatic tumor phenotype.

### High expression of JMJD6 in human breast tumors is associated with a worse prognosis of Myc-high tumors

**Fig. 10:**
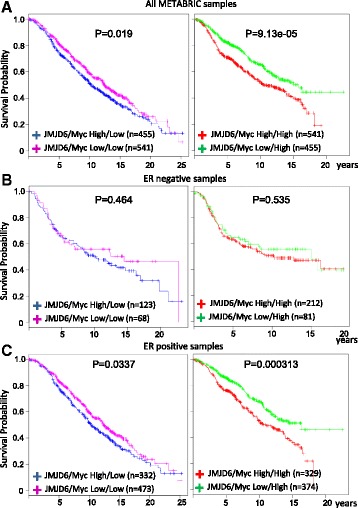
High expression of JMJD6 and Myc shows the worst prognosis for human mammary gland tumors. **a** Kaplan-Meier survival curves are shown for the high versus low expression of JMJD6 in the presence of low (*left panel*) or high (*right panel*) expression of Myc. METABRIC database (~2000 patients) was used to perform this analysis. Analysis was performed as in **a** for **b** ER-negative or **c** ER-positive mammary gland tumors

The in vitro and in vivo results presented above suggest that the combination of high expression of Myc and JMJD6 might be associated with a poor clinical prognosis. To determine whether this might be true for human breast cancers, we performed an in silico analysis using the METABRIC database that contains microarray data and clinical information on about 2000 breast cancer patients. All cases were divided into four subsets, consisting of high/low, high/high, low/high, and low/low of JMJD6/Myc expression. When we compared patients with high versus low JMJD6 expression, we observed a highly significant effect in samples with high Myc rather than low Myc expression (compare *p* values for left and right panels in Fig. 10a, c). We determined that high JMJD6 expression is associated with a poor prognosis for ER-positive breast cancer patients and not for ER-negative breast cancer (Fig. 10b), consistent with a previous report analyzing JMJD6 expression as a biomarker for poor prognosis in ER+ breast cancer [25]. Overall, this analysis predicts that JMJD6 gene expression may be a discriminating factor for survival of patients with high Myc expression in ER-positive breast cancer patients.

## Discussion

Our array CNV results of 8 GEM models of mammary cancer demonstrated that there are common genomic amplifications and losses in multiple models as well as alterations that are more pronounced in specific models. These data, consistent with prior reports, demonstrates that the evolution of genomic alterations during tumorigenesis in these models appears to depend upon the genetic event that initiates transformation. We focused on the MMTV-Myc model since Myc is overexpressed in about 30 % of human breast cancers and this model displays relatively few CNVs except for a prominent amplification of mouse distal chromosome 11.

We identified several candidate genes within this locus that could potentially cooperate with Myc and enhance tumorigenesis. Functional analyses determined that the epigenetic modifier JMJD6 cooperates with Myc to augment oncogenic transformation of normal mammary epithelial cells as well as to increase the aggressiveness and metastatic potential of Myc-driven tumors. Increased expression of JMJD6 resulted from the in vivo selection for the amplification of the distal region of chromosome 11 in Myc-overexpressing tumors. The chromosome 11 amplified locus is not exclusively specific for Myc-driven tumors but also has been found in mouse mammary gland tumors with overexpression of Erbb2, PyMT, or BRCA1 mutations (this study and [8, 13–16, 23]), which implicates JMJD6 as potentially augmenting tumorigenesis in other oncogenic pathways.

Myc activation in normal cells is not capable of promoting neoplastic transformation because of the overwhelming induction of cell death triggered by Myc protein expression above normal physiological levels [47–51]. The mechanisms of Myc-induced apoptosis have been studied very extensively, and involve multiple pathways of Myc-dependent cell death and proliferation. First, similar to other oncogenes, supraphysiological levels of Myc induce p19ARF (p14ARF in human) that sequesters MDM2 E3 ubiquitin ligase which leads to stabilization of p53 [33, 35]. Consistent with this finding, loss of p19ARF or p53 allows Myc to immortalize primary cells and synergize with Myc-induced tumorigenesis [52–54]. There is also evidence that ARF can limit Myc activity by direct protein-protein interaction independent of p53 and MDM2 [55, 56]. Therefore, ARF mediates its tumor suppressor activity through p53-dependent and -independent mechanisms to prevent Myc-stimulated proliferation and cellular transformation. In this study, we found that JMJD6 decreases Myc-induced apoptosis, at least in part, by suppressing p19ARF expression. It would be interesting to analyze the cooperation of JMJD6 with other oncogenes whose action is also restricted by ARF.

There are multiple functions assigned to the JMJD6 enzyme. It was first discovered as a histone arginine demethylase [36] and a lysine hydroxylase of histone and non-histone proteins [29, 30]. The function of JMJD6 as an epigenetic modifier of histone proteins has been widely debated in the literature. The unique property of JMJD6 is its ability to function as an arginine demethylase. Currently, it is the only known arginine demethylase that is capable of targeting both histone and non-histone proteins. In vitro studies have demonstrated the removal of monomethyl groups from histone arginine residues as well as symmetric and asymmetric methyl groups [32, 57]. That implies that JMJD6 may function as a transcriptional activator and transcriptional repressor, as H4R3me2s (symmetric) is a repressive mark [37, 38] and H4R3me2a (asymmetric) methylation is a mark of active chromatin [37, 39, 40]. Consistent with this notion, diminished expression of p19ARF in cells with high JMJD6 levels presented in this study was accompanied by a decrease in histone H4R3me2a modification (a marker of active chromatin) in the p19 promoter. This was associated with reduced p53 expression and reduced apoptosis.

Consequences of lysine hydroxylation of non-histone proteins may be variable. For example, when JMJD6 hydroxylates splice factor U2AF65 [30, 58], it changes VegfR1 mRNA splicing in endothelial cells in favor of the angiogenesis promoting long form of the protein [31, 59]. Another example of non-histone protein modification by JMJD6 has recently identified its ability to hydroxylate Lys382 in p53 protein in the colorectal cancer cell line HCT116 [26]. This modification does not affect p53 protein stability but rather inhibits its transcriptional activity. This is, however, not the case for normal mouse epithelial cells used in this study, as we clearly see decreased p53 at the protein level (Fig. 6). We are currently exploring the possibility that JMJD6 also induces p53 hydroxylation in non-tumorigenic mouse and human cells.

Several publications identified Brd4 as a binding partner of JMJD6 in different cell types [32, 60, 61]. Brd4 is classified as a histone code “reader” that functions by recognition of acetylated histones and recruiting epigenetic modifiers to the target gene [62]. Assembly of the Brd4-JMJD6 complex in distant enhancers of target genes stimulates histone H4R3me2(s) demethylation as well as cap removal from 7SKsnRNA, promoting transcriptional activation regulated by anti-pause enhancers [32]. Interestingly, Myc protein was also implicated in the transcriptional pause release of active genes [63]. When Myc levels were increased, similar to Myc-amplified tumors, Myc also occupied distant enhancers of actively transcribed genes [64] which raises the question of whether JMJD6 amplification in MMTV-Myc tumors is necessary for the overall increase of transcriptional activation by Myc. Further experimentation will be required to fully understand the extent of Myc-JMJD6 cooperation.

Elevated levels of JMJD6 protein and mRNA were reported for several tumor types, breast, lung, and colon cancers [25–27]. For colon cancer, elevated JMJD6 expression positively correlated with depth of invasion, lymph node metastasis, and advanced tumor node metastasis stage [27]. In agreement with this, we observed a causal role of JMJD6 in dramatically promoting metastasis in Myc-driven tumors (Fig. 9).

Myc is a proto-oncogene that triggers tumorigenesis by increasing cellular proliferation. However, paradoxically, it has been reported that in breast cancer cells, it can act as a metastasis suppressor, decreasing cell invasion in vitro and reducing metastatic burden in mice in vivo [46]. In our experiments, overexpression of JMJD6 in MMTV-Myc tumor cells was able to dramatically increase the number of metastatic nodules in lungs of mice after tail vein injection, as well as invasion and migration of these cells in vitro. In addition, analysis of a large cohort of breast cancer patients (more than 2000) revealed that high JMJD6 expression in human breast cancer with high Myc results in a lower overall survival presumably due to a higher metastatic burden (Fig. 10). This is consistent with a previous report that demonstrated that high JMJD6 was associated with a worse prognosis in ER+ breast cancer [25]. ERα function is regulated in part by methylation of R260, which is required for estrogen-induced complex formation of ERα with Src and PI3K and activation of the Akt pathway [65]. JMJD6 demethylates this R260me2a after treatment with estrogen [57], which implies a compromised, non-genomic function of ERα. However, it remains unclear how JMJD6 expression leads to a worse prognosis in ER+ patients.

## Conclusions

Our studies have revealed important cooperativity between JMJD6 and the Myc proto-oncogene and possibly other types of oncogene-driven breast cancers. These results suggest that JMJD6 protein expression may be used as a prognostic biomarker in future studies. Additionally, since Myc is overexpressed in many breast cancers but not successfully targeted by drugs, inhibiting JMJD6 function may provide a means of inhibiting Myc-dependent tumorigenesis.

## Methods

### DNA copy number

Genomic DNA was extracted from tumors or spleen (as reference) from 8 GEM models of mammary cancer using DNeasy Blood and Tissue kit (Qiagen, USA). Five hundred nanograms of reference or test sample DNA was labeled with Cy3 and Cy5 using Enzo CGH labeling kit (Enzo Life Sciences, USA) according to the manufacturer’s instructions. The labeled DNAs were purified with Amicon Ultra-0.5, 30Kd filters and hybridized to Agilent 44K CGH arrays for 44 h at 65 °C in a rotating microarray hybridization chamber and then washed with buffer 1 for 5 min at room temperature and buffer 2 for 1 min at 37 °C. After a brief rinse in acetonitrile and Agilent stabilizer (Agilent 5185–5979), the arrays were scanned in an array holder covered with the ozone barrier using the Agilent DNA microarray scanner G2539A. The array features were extracted using the Agilent Feature Extraction v10.7. The copy numbers were analyzed using Agilent Genomic Workbench 5.0 program and Nexus Copy Number software (BioDiscovery, USA).

Data have been deposited to the Gene Expression Omnibus (accession number GSE75331). The reviewer access url is http://www.ncbi.nlm.nih.gov/geo/query/acc.cgi?token=yxclqmqynzadxgx&acc=GSE75331.

#### Gene expression microarray

Total RNA (1 μg) was reverse transcribed with a T7-oligo(dT) primer and labeled with biotin using the Affymetrix One Cycle Target Labeling kit (Affymetrix, USA), following the manufacturer’s protocol. RNA was then labeled and hybridized to the mouse genome 430A 2.0 GeneChip (Affymetrix, USA) and scanned on an Affymetrix GeneChip scanner 3000. Data were collected using Affymetrix GCOS software. Data analysis was described in Zhu et al., 2011 [66]. Data have been deposited to the Gene Expression Omnibus (accession number GSE23938).

### Cell lines

NMuMG epithelial cells were purchased from ATCC. MMTV-Myc mammary gland tumor cell lines Myc83 and 88CT1 were obtained from Dr. R. Dickson (Georgetown University). Cells were cultured in DMEM (high glucose) tissue culture media, supplemented with 10 % fetal bovine serum (Life Technologies, USA) and penicillin/streptomycin. Cultures were incubated in humidified air containing 5 % CO_2_ at 37 °C. Cells in nutrient deprivation experiments were cultured in DMEM glucose-free, glutamine-free media (Life Technologies, USA), supplemented with 10 % dialyzed FBS (dialyzed against 3 changes of 100 volumes of PBS) [67]. The remaining components (glucose or glutamine) were added separately. 4-hydroxytamoxifen was purchased from Sigma and used at a final concentration of 150 nM. Etoposide (Sigma, USA) was added for 24 h at a final concentration of 100 μM.

### Plasmids

The lentiviral vectors pLenti6.2 expressing LacZ-V5 or JMJD6-V5 were described by Lee et al. [25] and were a kind gift of Dr. K. Desai (National Institute of Biomedical Genomics, India). The pre-designed lentiviral shRNA vectors for knock-down of JMJD6, TK1, Tnrc6c, Sumo2, FBF1, Birc5, and Ube2o were cloned into MISSION vector with puromycin resistance and purchased from Sigma. PBabePuro-MycER^TM^ used for Myc induction in cells overexpressing JMJD6 or LacZ was a gift from Dr. G. Evan (UCSF), and pBabeHygro-MycER^TM^ used with shRNA knock-down experiments was a gift from Dr. Y. Lazebnik (Cold Spring Harbor laboratory). Retroviral vector pBabe-p19ARF was a gift from Dr. P. Johnson (NCI).

### Real-time quantitative PCR

Total RNA was isolated from cells by TRIzol extraction (Life Technology). One microgram of total RNA was reverse transcribed in a 50-μl reaction using TaqMan Reverse Transcription Reagents (Life Technologies, USA) according to manufacturer’s instructions. Five microliters of the resultant reaction was PCR amplified in a total volume of 20 μl for 40 cycles using an Applied Biosystems 7500 instrument. TaqMan probes were purchased from Life Technologies. Mouse Tbp (TATA binding protein) was used as a standard for normalization. The gene expression for p19ARF was also validated with the specific pre-designed probe Mm.PT.58.8388138 (Integrated DNA Technologies, USA) with SYBR Green qPCR master mix (Bio-Rad, USA). All reactions were performed in triplicate.

### Chromatin immunoprecipitation

For immunoprecipitation with anti-H4R3me2a antibody (Active Motif, USA) cells were fixed with 1 % formaldehyde (Sigma, USA) for 15 min at room temperature. For immunoprecipitation with anti-JMJD6 antibody (Millipore, USA), cells were first fixed with 2 mM disuccinimidyl glutarate (DSG) (ProteoChem, USA) for 45 min at room temperature, followed by two washes with cold PBS and additional fixation with 1 % formaldehyde for 15 min. ChIP assays were performed using a ChIP-IT High Sensitivity Kit from Active Motif (USA). Fixation was stopped by addition of 1/20 volume of stop buffer, and aliquots of 4 × 10^6^ cells were lysed in 400 μl of SDS-lysis buffer. DNA shearing was performed for a total of 16 min at high power setting in BIORUPTOR water bath sonicator (Diagenode, USA). Immunoprecipitation was performed with 1.4 μg of anti-JMJD6 or 10 μg of anti-H4R3me2a antibody or 40 μl of V5-agarose (Sigma) at 4 °C overnight. DNA protein complexes were collected with protein G agarose beads and washed according to manufacturer’s instructions. After elution and reversal of crosslinking, samples were treated with RNase A and proteinase K and purified with QIAQuick PCR columns. Five microliters of the eluted DNA was used for quantitative PCR analysis with SYBR Green qPCR master mix (Bio-Rad, USA). For mouse p19ARF, primers used [68] were forward primer 5′-TTCCAGGCCTTGCCATCTTCCTAT-3′ and reverse primer 5′-TGGTCTGGCTGCAGTAAAGTAGCA-3′. For human p14ARF, we used two pairs of primers: distal forward 5′-CATTTCTGAGGAAGGGCTACTT-3′ and distal reverse 5′-GCCACCTTTCTGTCTAGTATGG-3′, proximal forward 5′-TCGCCAAGACAACCATTCTAC-3′ and proximal reverse 5′-CGCTTCTTCCTCTTTCCTCTTC-3′. For amplification of mouse p16 promoter, the primers were forward 5′-CAGATTGCCCTCCGATGACTTC-3′ and reverse 5′-TGGACCCGCACAGCAAAGAAGT-3′. Primers for β-globin used as a positive control were provided by Millipore together with ChIP quality anti-JMJD6 antibody.

### Cell cytotoxicity assay

Cells were seeded at 10,000 cells per well in 96-well non-transparent plates. The next day, cells were treated with 150 nM 4-hydroxytamoxifen (4-OHT) to induce Myc or with equal volume of EtOH as a control. Twenty-four hour later, cells were washed twice with PBS and the media was changed for “stress media” that included either serum-free media, glucose-free media, glutamine-free media, or 100 μM etoposide. After 18–20 hours the percentage of dead cells relative to the total amount of cells was measured using the luminescent CytoTox-Glo cytotoxicity assay (Promega, USA). Briefly, 50 μl of CytoTox-Glo cytotoxicity reagent was added to each well, mixed and incubated for 15 min at room temperature. After measuring the luminescent signal, 50 μl of lysis reagent containing digitonin was added for another 15 min to lyse the remaining viable cells. After incubation, the second luminescence measurement was taken and the percentage of dead cells was calculated by dividing the first luminescence by second after subtraction of the background signal for media only. Each experiment was performed in triplicates and repeated 2–3 times.

The CytoTox-Glo assay measures the number of dead cells irrespective of how cells died. To confirm that majority of cell die from apoptosis after Myc induction, we repeated the experiments, performing FACS analysis after staining the cells with FITC-Annexin V and propidium iodide using the Apoptosis Detection Kit (BD Pharmingen, USA). The data are shown in Additional file 1: Figure S9.

### Migration and invasion assay

2 × 10^4^ cells expressing JMJD6 or LacZ control were placed into the top compartment of transwell Boyden chambers (8 μm, Corning, USA) in serum-free media. The lower compartment contained complete media with 10 % FBS. For the invasion assay, we used the same setting with Matrigel™-covered chambers prepared according to the manufacturer’s instructions (Corning, USA). After 24 h, migrated cells were fixed and stained with the Diff-Quik Stain Set (Dade Behring Inc., USA). The cells that migrated through the pores of the membrane were photographed and quantified using ImageJ software.

### Western blot analysis

Protein lysates were prepared in RIPA buffer and 20–50 μg of protein were fractionated in a 10 % SDS-PAGE gel and transferred to a PVDF membrane (Immobilon-P, Millipore). The membrane was blocked with 5 % milk (Bio-Rad Laboratories), probed with antibodies, followed by washing and incubation with HRP-conjugated secondary antibody (Pierce) and developed using the WesternBright ECL^TM^ reagent (Advansta, USA). Antibodies used were anti-V5 (Life Technologies, USA), anti-p53 (Becton Dickinson, USA), anti-Myc, anti-p53-Ser15, anti-p21 (Santa Cruz Biotechnology, USA), anti-Caspase3, anti-PARP, anti-Bmi1, anti-Bcl2, anti-Bcl-xL, anti-Snail, anti-Slug (Cell Signaling, USA), anti-Bcl-W (GeneTex, USA), anti-p19ARF, anti-JMJD6 (Abcam, USA), anti-vimentin, and anti-β-actin (Millipore, USA).

### Animal studies

Cells were trypsinized and washed twice with PBS, and 10^6^ cells in 100-μl PBS were surgically implanted into the #4 mammary fat pad of FVB/N mice (Charles River, USA). One week later, staples were removed and tumor volume was measured weekly with a caliper over 1 month. For the lung colonization assay, cells were prepared the same way and 5 × 10^5^ cells in 100-μl PBS were injected by tail vein into FVB/N mice. Three weeks later, mice were euthanized, lungs were formalin fixed and paraffin embedded, and sections were stained with hematoxylin-eosin solution.

All GEM models were described by Zhu et al. [66].

All mice were treated in accordance with the Guide for the Care and Use of Laboratory Animals (NIH publication no. 86–23, 1985) under an animal protocol (LCBG-063) approved by the IACUC of the National Cancer Institute (NCI).

### TUNEL assay

Lungs from mice were formalin fixed and embedded in paraffin, and three 5-μm step sections were obtained for each lung. Sections were deparaffinized by standard treatment with xylene and ethanol. The following procedure was performed using the ApopTag Peroxidase In Situ Apoptosis Detection Kit (Millipore, USA) according to the manufacturer’s instructions. Briefly, the tissue was pretreated with proteinase K (20 μg/ml), endogenous peroxidase was quenched by 3 % hydrogen peroxide for 5 min, and slides were incubated with TdT enzyme for 1 h at 37 °C. After washing, slides were incubated with anti-digoxigenin conjugate for 30 min at room temperature, washed and developed with peroxidase substrate. Slides were counterstained with methyl green and after dehydration mounted under glass coverslips using Permount media (Fisher Scientific, USA). Three photographs were taken from each slide and analyzed using ImageJ software.

### Immunofluorescence

Cells expressing JMJD6 or LacZ were grown on cover slips, fixed with 4 % paraformaldehyde for 15 min, and permeabilized with 0.2 % Triton X-100 for 4 min, and immunostaining was performed by 1-h incubation with anti-V5 antibody (Life Technologies, USA) followed by 30-min incubation with the secondary goat anti-mouse antibody, conjugated with Alexa Fluor 568 (Life Technologies, USA). Nuclei were counterstained with DAPI and images were taken with Nikon Eclipse E800 fluorescent microscope (Nikon).

### Statistical analysis

Statistical analysis was performed with GraphPad Prism5 software that shows mean values with 95 % confidence interval. The *p* values were calculated using Student’s *t* test (two tailed). The TaqMan data were analyzed using ΔΔCt method and presented as mean fold change ±SEM. The Pearson correlation coefficient for mouse mammary gland tumors was determined to assess the correlation of JMJD6 and p19ARF expressions.

The expression data for JMJD6 and Myc in human mammary gland tumors were obtained from the METABRIC [69]. For each gene, the binary expression values (high or low) were defined by dichotomizing continuous expression values using the median as the threshold. We used the two genes JMJD6 and Myc jointly to define four patient sub-groups: JMJD6 high/Myc low, JMJD6 low/Myc low, JMJD6 high/Myc high, and JMJD6 low/Myc high. To examine the effect of JMJD6, we compared the Kaplan-Meier curves between the two sub-groups JMJD6 high/Myc low versus JMJD6 low/Myc low. We also compared JMJD6 high/Myc high versus JMJD6 low/Myc high. *p* values were obtained using log-rank test.

## Additional files

Additional file 1: Figures S1–S9. Figure S1. The genome locus coordinates and number of genes in the minimal region of chromosome 11 amplicon in MMTV-Myc mammary gland tumors. Figure S2. CGH analysis of two cell lines derived from MMTV-Myc mammary gland umors. A. Chromosomal gains and losses in Myc83 and 88CT1 cell lines. Arrow indicates the chromosome 11 amplicon in Myc83 cells consistent with the original CNV observations made in Fig. 1 for MMTV-Myc tumors. 88CT1 cells do not contain this amplicon. B. RT-qPCR validation of higher expression of three genes contained within the amplified region in Myc83 compared to 88CT1 cells. Figure S3. Analysis of cell death induced by glucose deprivation or etoposide treatment in cells with knock-down of 6 genes contained within the amplified region of chromosome 11. Analysis was performed as in Fig. 3 using cells with stable expression of shRNAs targeting the FBF1, Ube2o, Birc5, TK1, Sumo2, and Tnrc6c genes. Originally, 5 shRNAs for each gene were tested and shRNAs that achieved more than a 50 % downregulation of the targeted gene were selected for further analysis. Myc was induced in NMuMG cells expressing pBabe-MycER^TM^ by the addition of 150 nM 4-OHT (MycON) for 24 h. Myc-induced cell death was tested after treatment with 100 μM etoposide for 20 h. Figure S4. Efficiency of JMJD6 knock-down. Western blot analysis of JMJD6 expression in NMuMG and Myc83 cell lines stably expressing two different shRNA vectors. Figure S5. Ectopic expression of LacZ-V5 and JMJD6-V5 in NMuMG cells. A. Cells were stained with anti-V5 antibody, followed by incubation with a secondary antibody conjugated with Alexa-568 and DAPI. The images show nuclear localization for JMJD6 and cytoplasmic localization for LacZ. B. Western blot analysis of cells with JMJD6 overexpression using anti-JMJD6 antibodies. Figure S6. JMJD6 does not alter c-Myc protein levels or localization of MycER™ fusion protein. A. Western blot analysis of NMuMG-MycER™ cells expressing LacZ or JMJD6 with anti-Myc antibody after addition of 4-OHT. Left panel shows MycER™ fusion protein and right panel shows endogenous c-Myc protein in cells expressing LacZ or JMJD6. B. Immunofluorescent staining (anti-c-Myc antibody) of NMuMG-MycER™ cells expressing JMJD6 after treatment with 4-OHT (MycON) or ethanol (MycOFF) for 24 h. Figure S7. Reverse transcription-qPCR analysis of JMJD6 and p19ARF expression in mouse MMTV-Myc mammary gland tumors. RNA isolated from mammary gland tumors was processed as described in the “Methods” section. The ΔCT values for JMJD6 and p19ARF were normalized to mouse Tbp housekeeping gene expression. Figure S8. JMJD6 does not bind to p16 promoter in NMuMG cells. Primers for mouse p16 gene promoter were used as a negative control after chromatin immunoprecipitation with anti-JMJD6 antibody described in Fig. 7. Figure S9. Wild-type JMJD6, but not its mutant form, suppresses Myc-induced apoptotic cell death. The experiments were performed as described in Fig. 4. The percentage of dead cells was measured by FACS analysis of cells stained with Annexin V and propidium iodide. (PPTX 2558 kb)
