# Supplementary material for: The epigenetic modifier JMJD6 is amplified in mammary tumors and cooperates with c-Myc to enhance cellular transformation, tumor progression, and metastasis
Source: Clin Epigenetics. 2016 Apr 14;8:38. doi: 10.1186/s13148-016-0205-6 (PMC4831179; doi:10.1186/s13148-016-0205-6)
Supplement: Additional file 1: Figures S1–S9. — Figure S1. The genome locus coordinates and number of genes in the minimal region of chromosome 11 amplicon in MMTV-Myc mammary gland tumors. Figure S2. CGH analysis of two cell lines derived from MMTV-Myc mammary gland umors. A. Chromosomal gains and losses in Myc83 and 88CT1 cell lines. Arrow indicates the chromosome 11 amplicon in Myc83 cells consistent with the original CNV observations made in Fig. 1 for MMTV-Myc tumors. 88CT1 cells do not contain this amplicon. B. RT-qPCR validation of higher expression of three genes contained within the amplified region in Myc83 compared to 88CT1 cells. Figure S3. Analysis of cell death induced by glucose deprivation or etoposide treatment in cells with knock-down of 6 genes contained within the amplified region of chromosome 11. Analysis was performed as in Fig. 3 using cells with stable expression of shRNAs targeting the FBF1, Ube2o, Birc5, TK1, Sumo2, and Tnrc6c genes. Originally, 5 shRNAs for each gene were tested and shRNAs that achieved more than a 50 % downregulation of the targeted gene were selected for further analysis. Myc was induced in NMuMG cells expressing pBabe-MycERTM by the addition of 150 nM 4-OHT (MycON) for 24 h. Myc-induced cell death was tested after treatment with 100 μM etoposide for 20 h. Figure S4. Efficiency of JMJD6 knock-down. Western blot analysis of JMJD6 expression in NMuMG and Myc83 cell lines stably expressing two different shRNA vectors. Figure S5. Ectopic expression of LacZ-V5 and JMJD6-V5 in NMuMG cells. A. Cells were stained with anti-V5 antibody, followed by incubation with a secondary antibody conjugated with Alexa-568 and DAPI. The images show nuclear localization for JMJD6 and cytoplasmic localization for LacZ. B. Western blot analysis of cells with JMJD6 overexpression using anti-JMJD6 antibodies. Figure S6. JMJD6 does not alter c-Myc protein levels or localization of MycER™ fusion protein. A. Western blot analysis of NMuMG-MycER™ cells expressing LacZ or JMJD6 with anti-Myc antibo [file 13148_2016_205_MOESM1_ESM.pptx]

## Slide 1
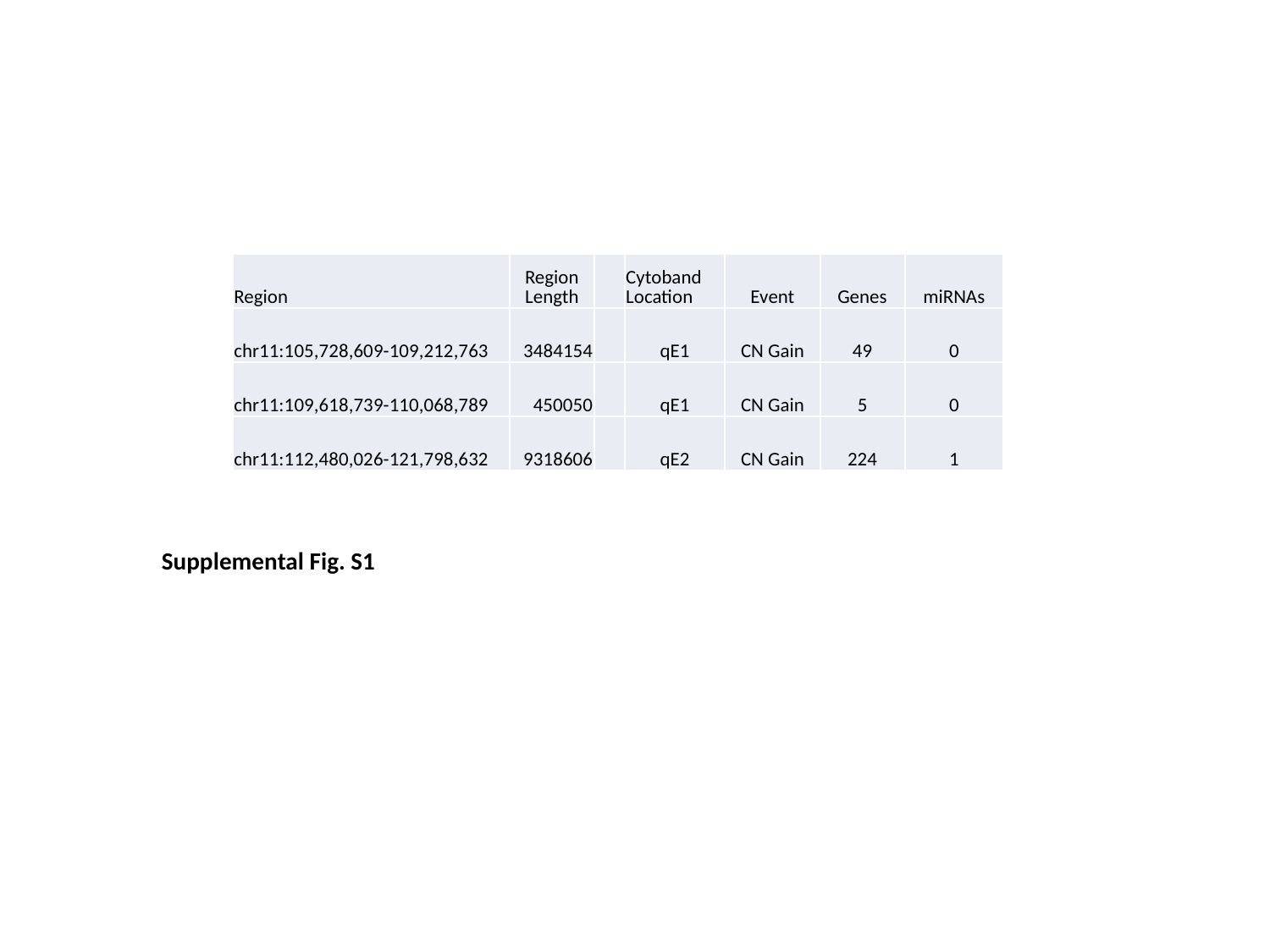

| Region | Region Length | | Cytoband Location | Event | Genes | miRNAs |
| --- | --- | --- | --- | --- | --- | --- |
| chr11:105,728,609-109,212,763 | 3484154 | | qE1 | CN Gain | 49 | 0 |
| chr11:109,618,739-110,068,789 | 450050 | | qE1 | CN Gain | 5 | 0 |
| chr11:112,480,026-121,798,632 | 9318606 | | qE2 | CN Gain | 224 | 1 |
Supplemental Fig. S1

## Slide 2
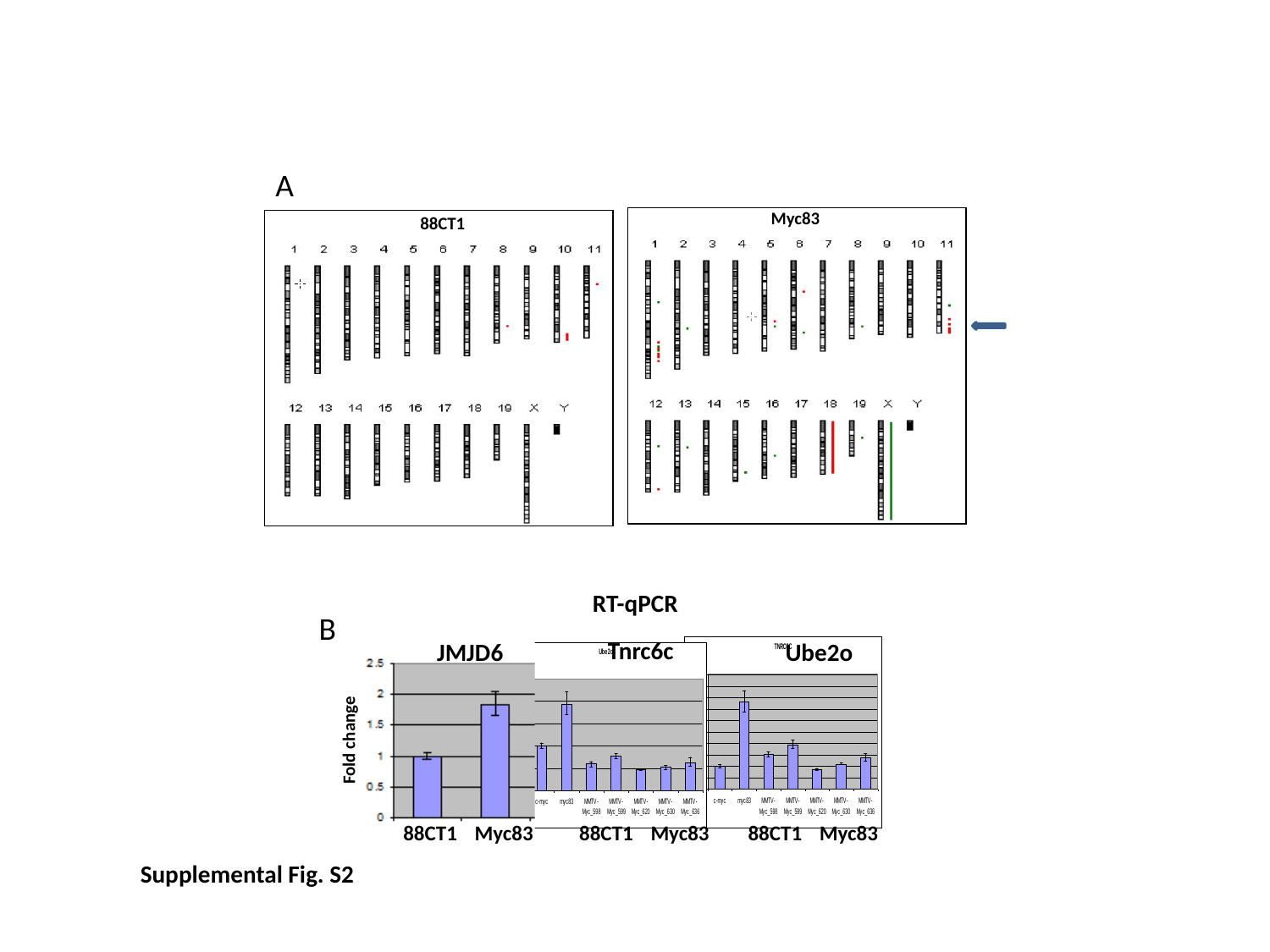

A
Myc83
88CT1
RT-qPCR
B
Tnrc6c
JMJD6
Ube2o
Fold change
88CT1
Myc83
88CT1
Myc83
88CT1
Myc83
Supplemental Fig. S2

## Slide 3
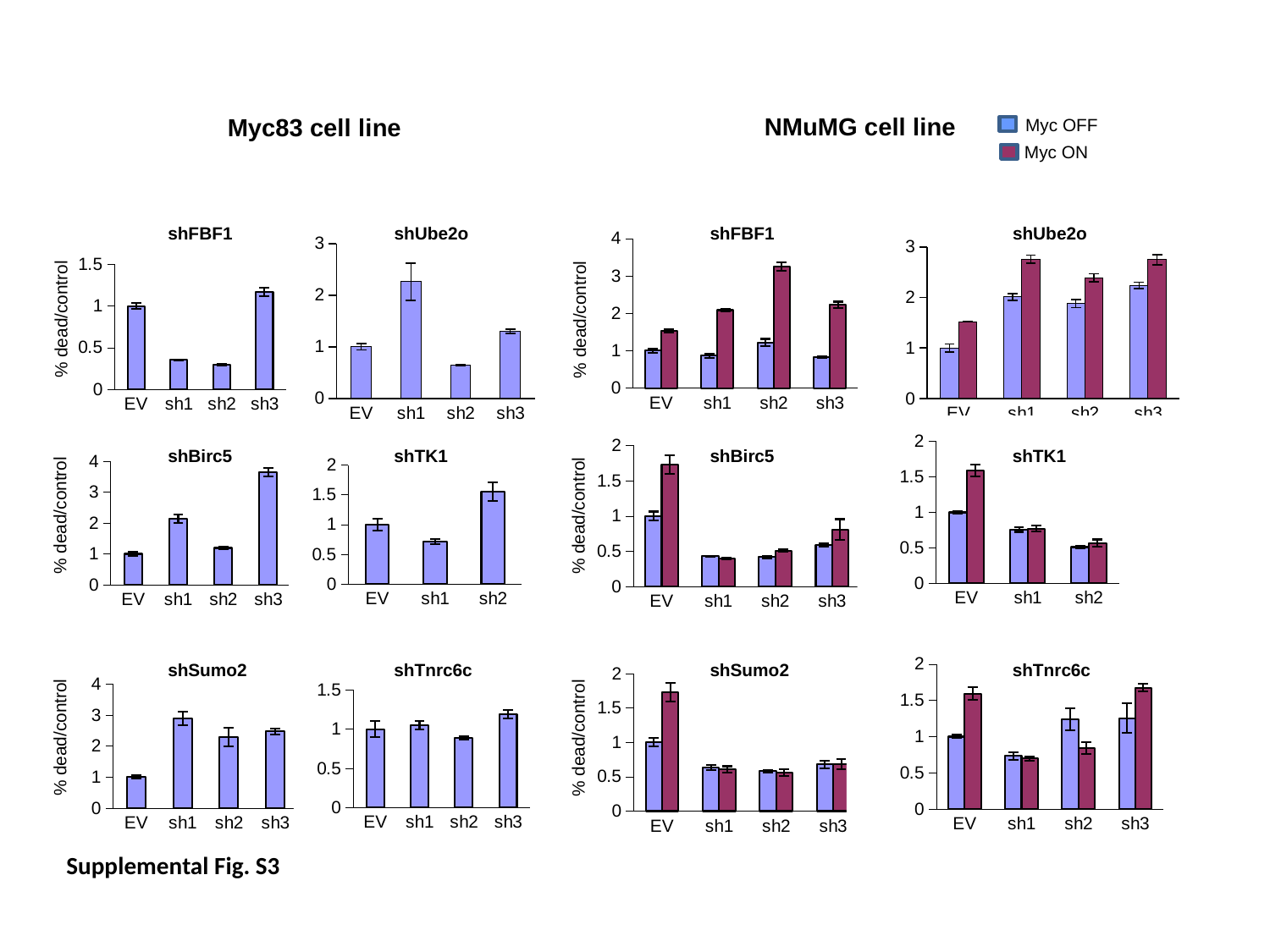

NMuMG cell line
Myc83 cell line
Myc OFF
Myc ON
### Chart
| Category | MycOFF | MycON |
|---|---|---|
| EV | 0.9999281193059222 | 1.5353983810646883 |
| sh1 | 0.8672608629071081 | 2.084293074001266 |
| sh2 | 1.219761580525719 | 3.245424277762071 |
| sh3 | 0.8312881595228988 | 2.2284827502509366 |shUbe2o
shFBF1
shUbe2o
shFBF1
### Chart
| Category | |
|---|---|
| EV | 1.0 |
| sh1 | 2.259343877045546 |
| sh2 | 0.6457043088739054 |
| sh3 | 1.2959733358652499 |
### Chart
| Category | MycOFF | MycON |
|---|---|---|
| EV | 1.0 | 1.522058403481626 |
| sh1 | 2.0115066403356523 | 2.7584974102578332 |
| sh2 | 1.8799324675649824 | 2.3894857859498044 |
| sh3 | 2.2392336054758264 | 2.7461252581830804 |
### Chart
| Category | |
|---|---|
| EV | 1.0000992450340789 |
| sh1 | 0.3537312191807002 |
| sh2 | 0.296632368298961 |
| sh3 | 1.1632353549768377 |% dead/control
% dead/control
### Chart
| Category | MycOFF | MycON |
|---|---|---|
| EV | 1.0000132429681636 | 1.5938636835760276 |
| sh1 | 0.7563916852024172 | 0.7673321371068246 |
| sh2 | 0.5108356901462234 | 0.5683864364866177 |
### Chart
| Category | MycOFF | MycON |
|---|---|---|
| EV | 1.0002906202920525 | 1.7304043180697708 |
| sh1 | 0.42970859838117476 | 0.4005234795233486 |
| sh2 | 0.4180955015927194 | 0.5109277799262157 |
| sh3 | 0.5953835156041493 | 0.8094052064360182 |shTK1
shTK1
shBirc5
shBirc5
### Chart
| Category | |
|---|---|
| EV | 1.0000751171974647 |
| sh1 | 2.1348723286944153 |
| sh2 | 1.1926999493136565 |
| sh3 | 3.6493021533978602 |
### Chart
| Category | |
|---|---|
| EV | 1.000001602621965 |
| sh1 | 0.7117970199740972 |
| sh2 | 1.5523896107855428 |% dead/control
% dead/control
### Chart
| Category | MycOFF | MycON |
|---|---|---|
| EV | 1.0000132429681636 | 1.5938636835760276 |
| sh1 | 0.7344062240540897 | 0.696357703182843 |
| sh2 | 1.2352511502971413 | 0.8452358364081429 |
| sh3 | 1.2546819964396618 | 1.6741846574502708 |shSumo2
shTnrc6c
shSumo2
shTnrc6c
### Chart
| Category | MycOFF | MycON |
|---|---|---|
| EV | 1.0002906202920525 | 1.7304043180697708 |
| sh1 | 0.6373512943741254 | 0.6035500520796458 |
| sh2 | 0.5804048106456301 | 0.5627124745992377 |
| sh3 | 0.6778408461679748 | 0.6862135398355139 |
### Chart
| Category | |
|---|---|
| EV | 1.0000751171974647 |
| sh1 | 2.8997894251663205 |
| sh2 | 2.28521649383022 |
| sh3 | 2.4691497431361595 |
### Chart
| Category | |
|---|---|
| EV | 1.000001602621965 |
| sh1 | 1.0518222472725771 |
| sh2 | 0.888551785191393 |
| sh3 | 1.1920201689690897 |% dead/control
% dead/control
Supplemental Fig. S3

## Slide 4
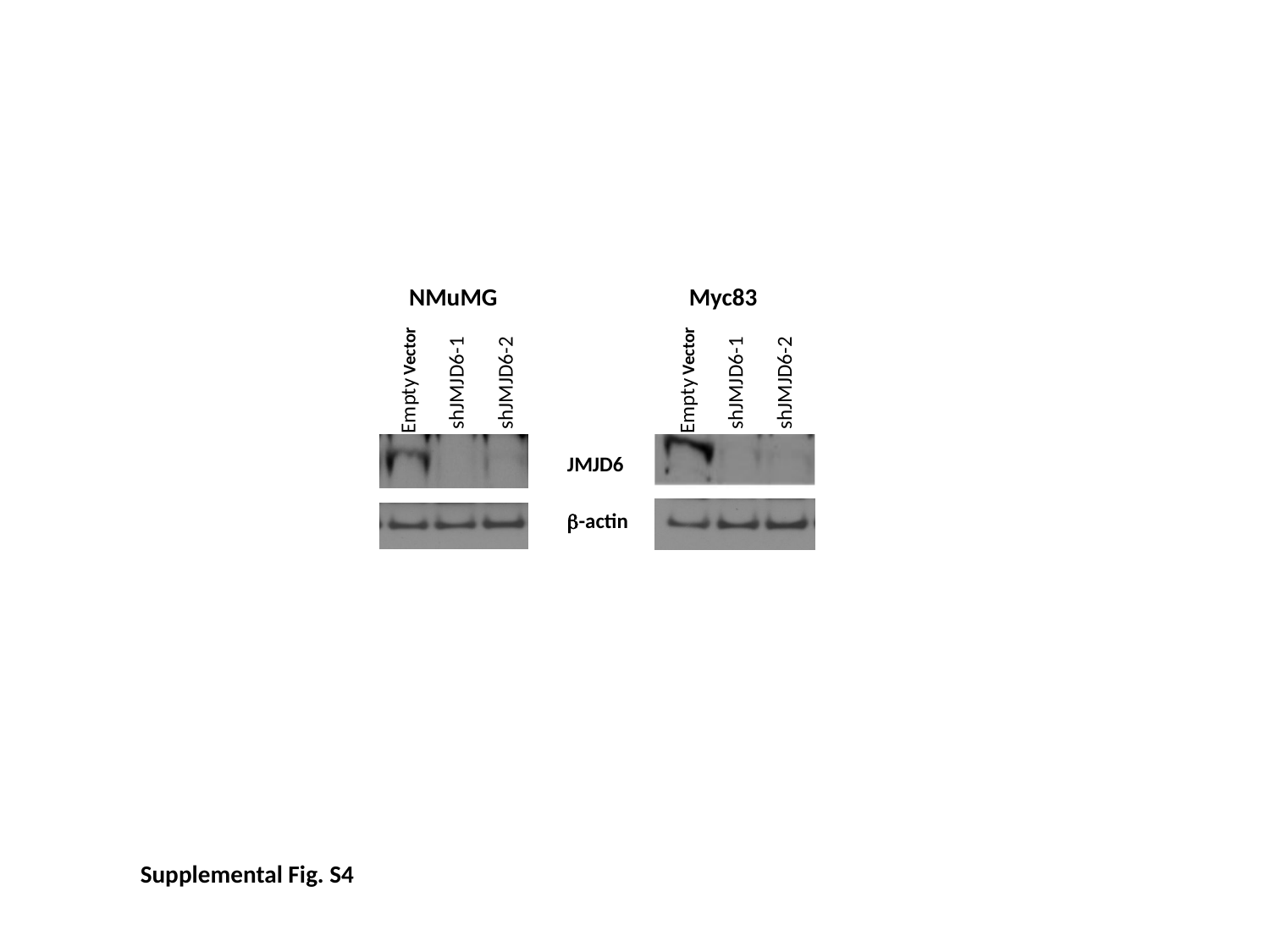

NMuMG
Myc83
Empty Vector
Empty Vector
shJMJD6-1
shJMJD6-2
shJMJD6-1
shJMJD6-2
JMJD6
b-actin
Supplemental Fig. S4

## Slide 5
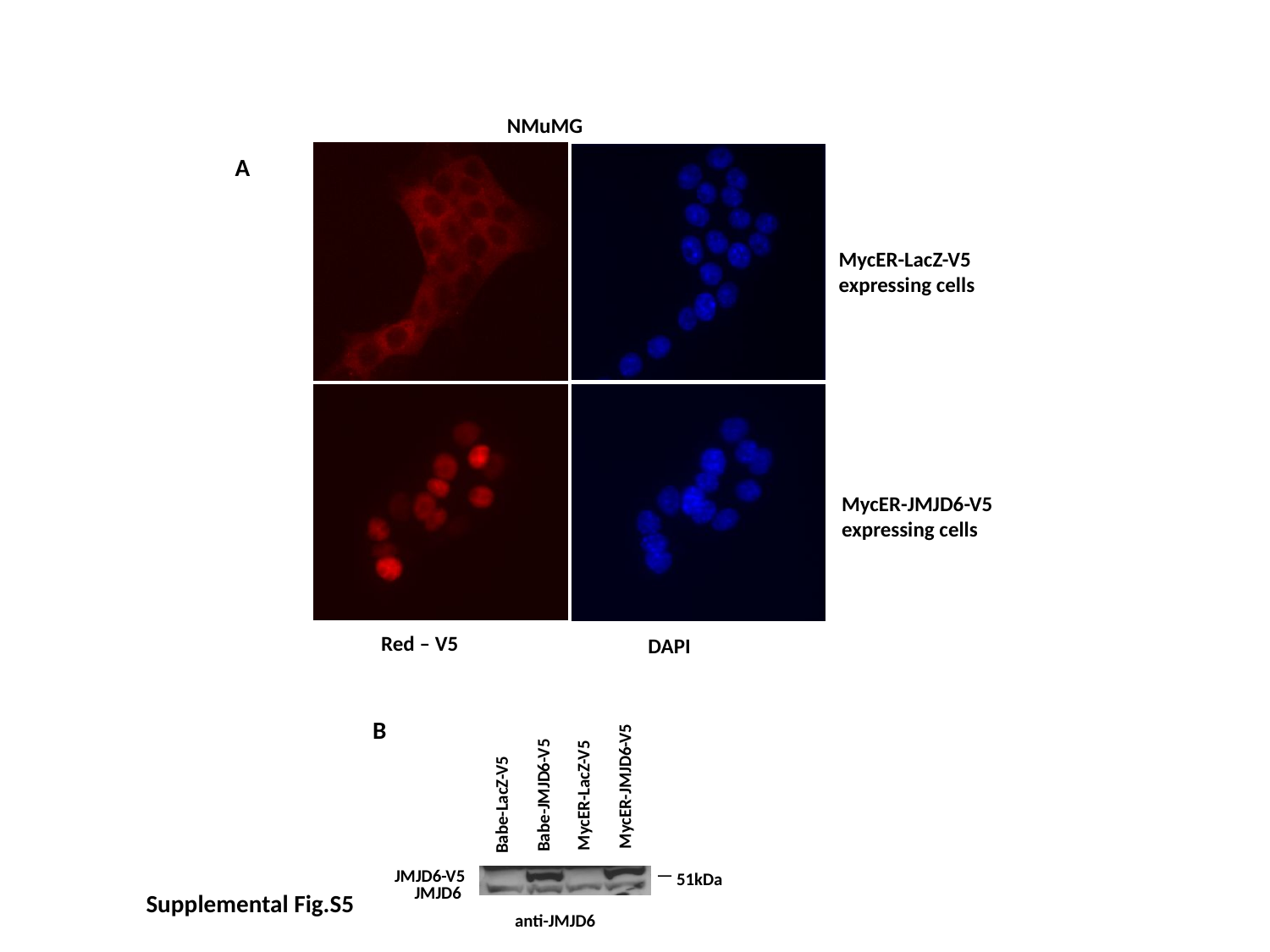

NMuMG
A
MycER-LacZ-V5
expressing cells
MycER-JMJD6-V5
expressing cells
Red – V5
DAPI
B
MycER-JMJD6-V5
Babe-JMJD6-V5
MycER-LacZ-V5
Babe-LacZ-V5
JMJD6-V5
51kDa
JMJD6
Supplemental Fig.S5
anti-JMJD6

## Slide 6
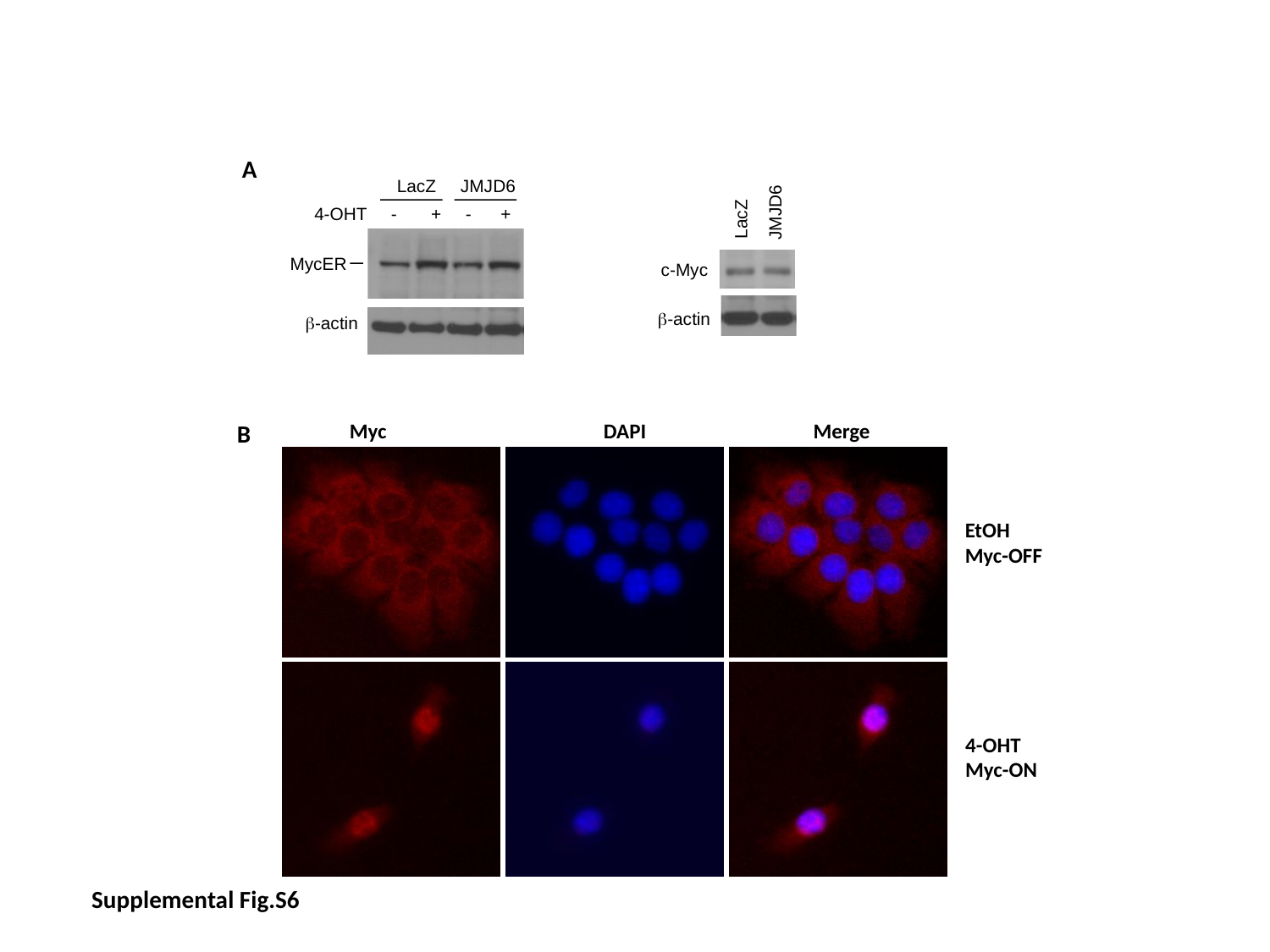

A
LacZ
JMJD6
JMJD6
4-OHT - + - +
LacZ
MycER
c-Myc
b-actin
b-actin
Myc
DAPI
Merge
B
EtOH
Myc-OFF
4-OHT
Myc-ON
Supplemental Fig.S6

## Slide 7
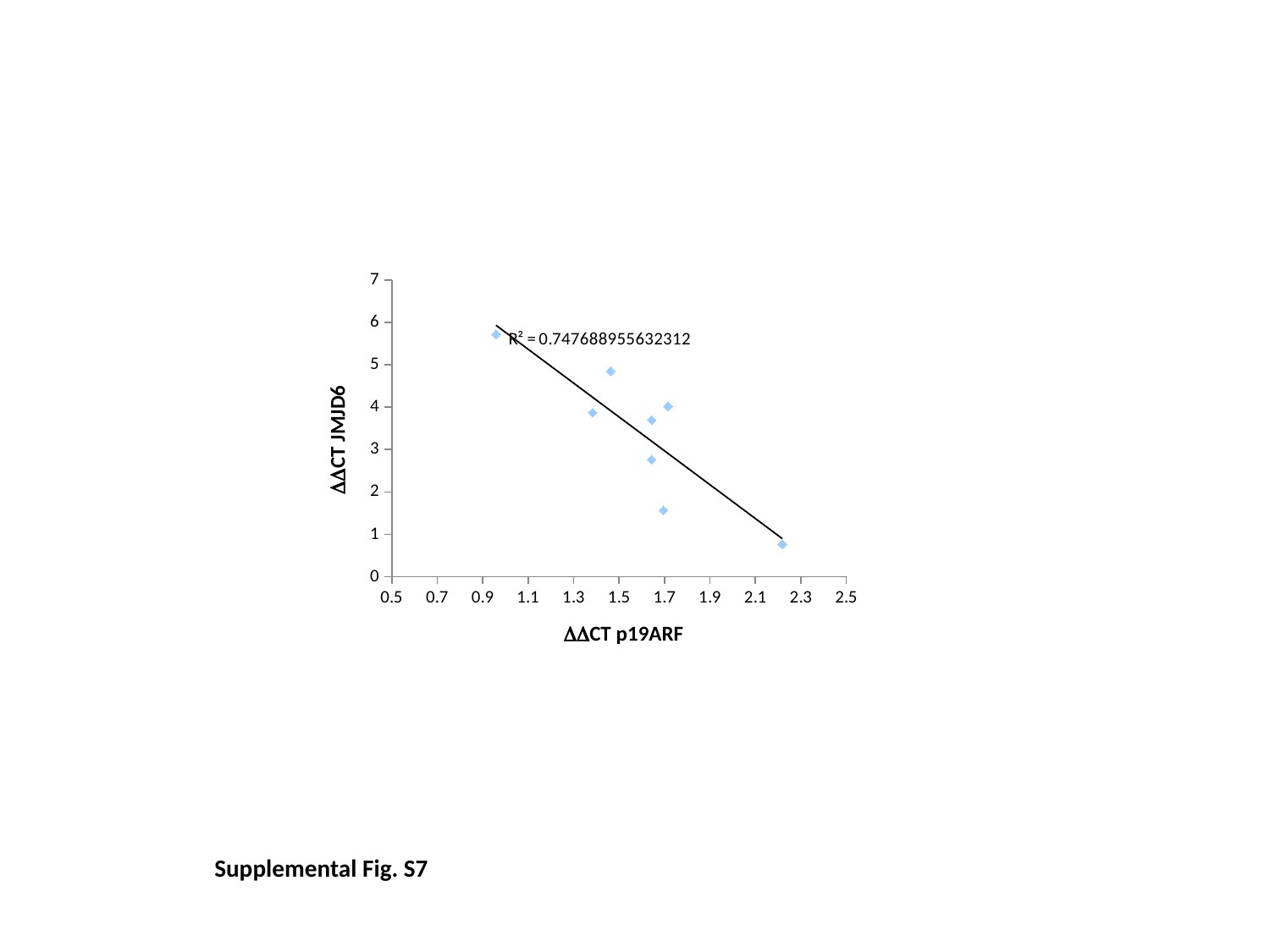

### Chart
| Category | |
|---|---|DDCT JMJD6
DDCT p19ARF
Supplemental Fig. S7

## Slide 8
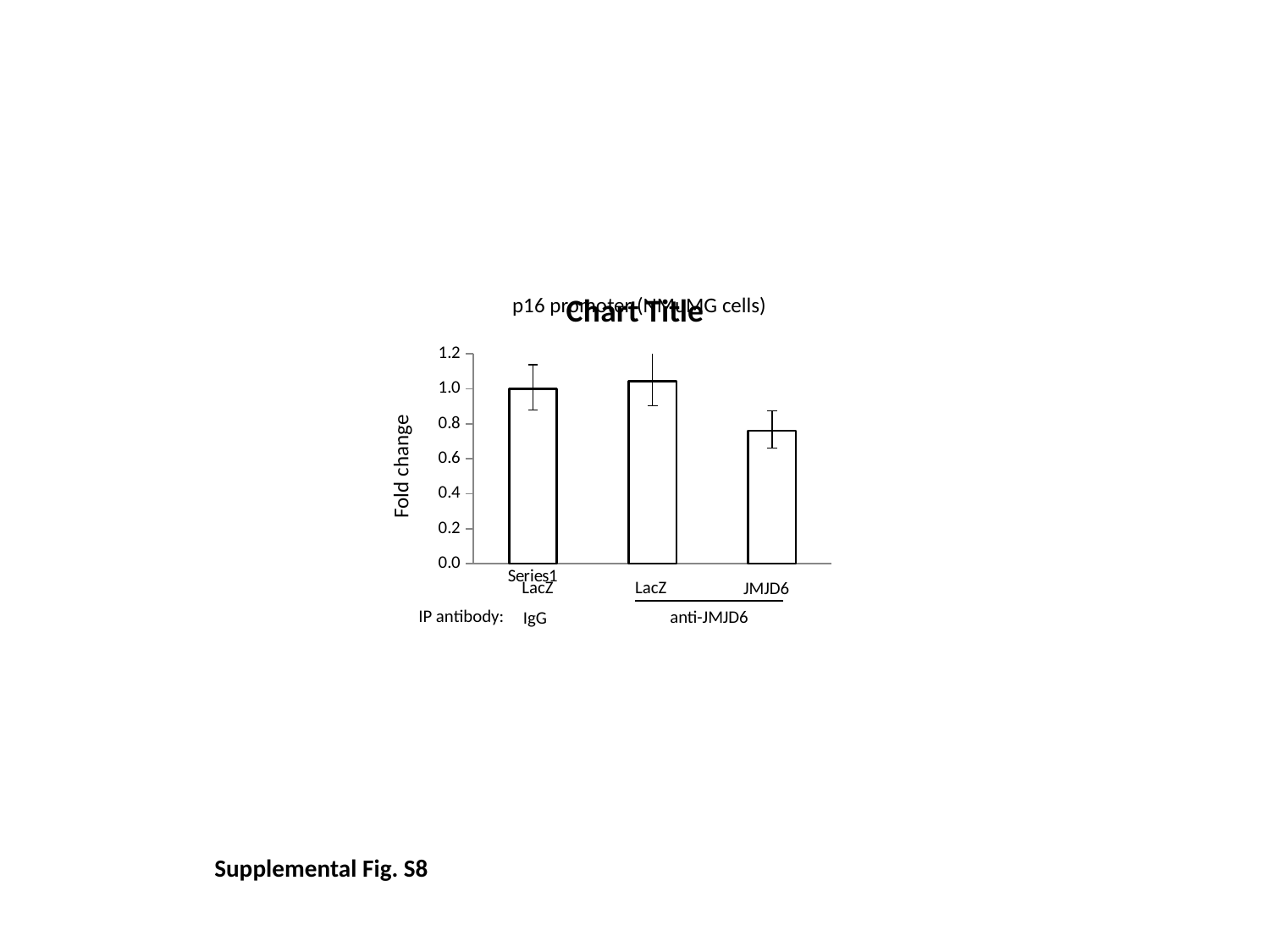

### Chart:
| Category | |
|---|---|
| | 1.0 |
| | 1.0442811137018015 |
| | 0.7604354274662042 |p16 promoter (NMuMG cells)
Fold change
 LacZ
 LacZ
JMJD6
IP antibody:
anti-JMJD6
IgG
Supplemental Fig. S8

## Slide 9
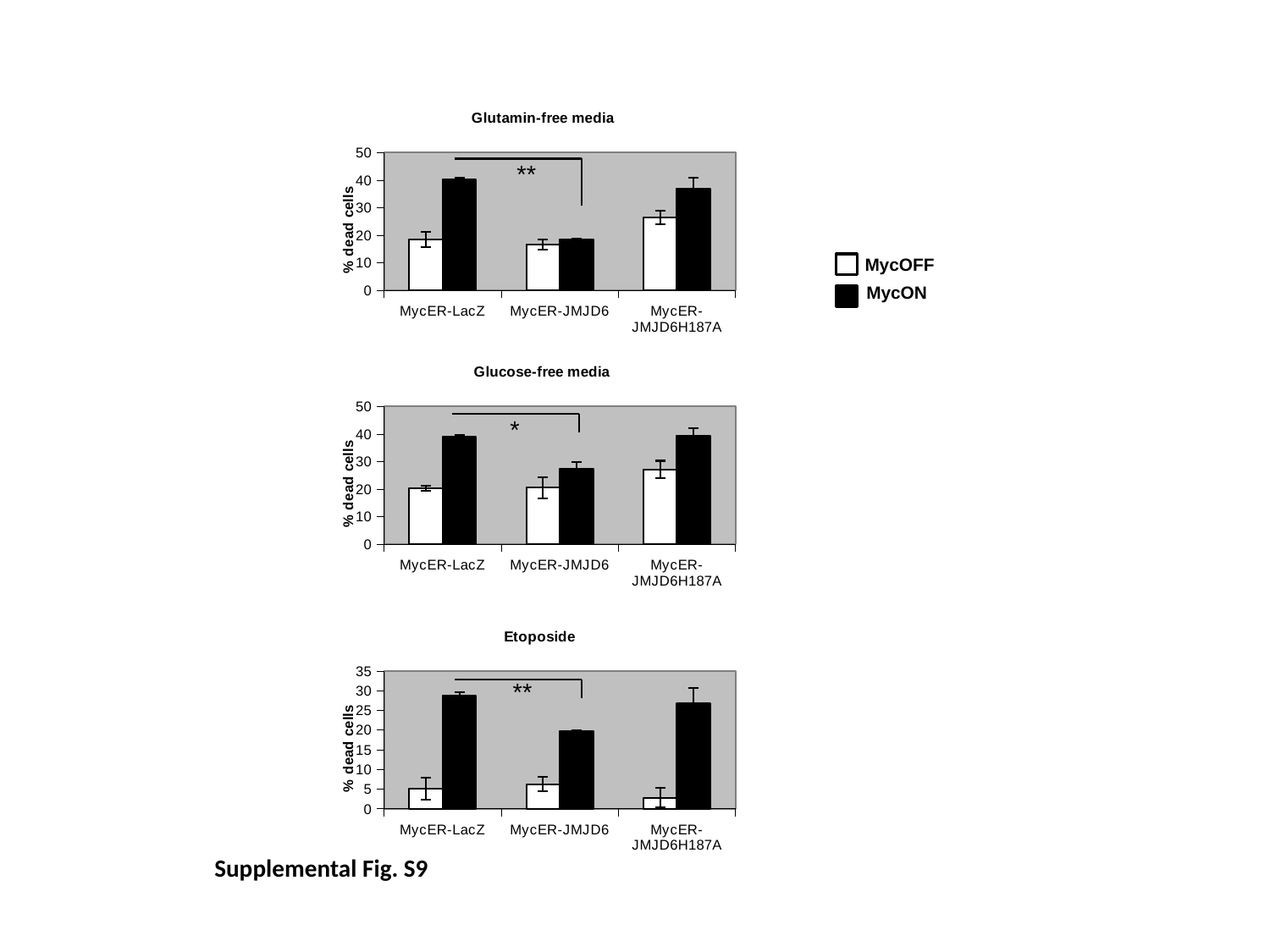

### Chart: Glutamin-free media
| Category | MycOFF | MycON |
|---|---|---|
| MycER-LacZ | 18.58 | 40.16 |
| MycER-JMJD6 | 16.725 | 18.46 |
| MycER-JMJD6H187A | 26.505 | 36.98 |**
MycOFF
MycON
### Chart: Glucose-free media
| Category | MycOFF | MycON |
|---|---|---|
| MycER-LacZ | 20.3 | 39.0 |
| MycER-JMJD6 | 20.55 | 27.45 |
| MycER-JMJD6H187A | 27.1 | 39.45 |*
### Chart: Etoposide
| Category | MycOFF | MycON |
|---|---|---|
| MycER-LacZ | 5.05 | 28.7 |
| MycER-JMJD6 | 6.25 | 19.65 |
| MycER-JMJD6H187A | 2.8 | 26.7 |**
Supplemental Fig. S9
